# Growth Arrest-Specific 6 Protein in Patients with Sjögren Syndrome: Determination of the Plasma Level and Expression in the Labial Salivary Gland

**DOI:** 10.1371/journal.pone.0139955

**Published:** 2015-10-07

**Authors:** Chen-Hung Chen, Hsiang-Cheng Chen, Chi-Ching Chang, Yi-Jen Peng, Chien-Hsing Lee, Yi-Shing Shieh, Yi-Jen Hung, Yuh-Feng Lin

**Affiliations:** 1 Graduate Institute of Clinical Medicine, Taipei Medical University, Taipei, Taiwan; 2 Division of Rheumatology, Immunology and Allergy, Department of Internal Medicine, Taipei Tzu Chi Hospital, Buddhist Tzu Chi Medical Foundation, School of Medicine, Tzu Chi University, Hualien, Taiwan; 3 Division of Rheumatology, Immunology and Allergy, Department of Internal Medicine, Tri-Service General Hospital, National Defense Medical Center, Taipei, Taiwan; 4 Division of Rheumatology, Immunology and Allergy, Department of Internal Medicine, Taipei Medical University Hospital, Taipei, Taiwan; 5 Department of Pathology, Tri-Service General Hospital, National Defense Medical Center, Taipei, Taiwan; 6 Division of Endocrinology and Metabolism, Department of Internal Medicine, Tri-Service General Hospital, National Defense Medical Center, Taipei, Taiwan; 7 School of Dentistry, National Defense Medical Center, Department of Oral Diagnosis and Pathology, Tri-Service General Hospital, Taipei, Taiwan; 8 Division of Nephrology, Department of Medicine, Shuang Ho Hospital, Taipei Medical University, New Taipei City, Taiwan; Instituto Nacional de Ciencias Medicas y Nutricion Salvador Zubiran, MEXICO

## Abstract

**Aims:**

Growth arrest-specific protein 6 (Gas6) is a vitamin K-dependent protein expressed by endothelial cells and leukocytes that are involved in cell survival, migration, and proliferation in response to inflammatory processes. The aim of this study was to assess the implications of Gas6 in Sjögren syndrome (SS) and its expression in the labial salivary gland.

**Methods and Results:**

A total of 254 adults, including 159 with primary Sjögren syndrome (pSS), 34 with secondary Sjögren syndrome (sSS), and 61 normal controls, were recruited. Plasma Gas6 concentrations were determined, and Gas6 expressions in labial salivary gland (LSG) tissues from controls and pSS and sSS patients were also evaluated. Plasma Gas6 concentrations were significantly lower among patients with pSS than normal controls (13.5 ± 8.6 *vs*. 19.9 ± 13.4 ng/ml, *p* < 0.001). There were, however, no significant differences in plasma Gas6 levels between pSS and sSS patients (13.5 ± 8.6 *vs*. 16.9 ± 11.2 ng/ml, *p* = 0.068). In multivariate logistic regression analysis, after adjustment for white blood cell count, hemoglobin level, platelet count, lymphocyte count, and C3 and C4 levels, lower plasma Gas6 concentrations were significantly associated with an increased risk of SS. Moreover, by using a semi-quantitative scale to evaluate Gas6 expression in LSG tissues, Gas6 expression was found to be markedly lower in LSG tissues from pSS patients than in tissues from normal controls.

**Conclusions:**

Decreased plasma Gas6 concentration and LSG expression were associated with pSS. As such, Gas6 may represent a novel independent risk factor for pSS, with a potential role in salivary gland inflammation and dysfunction.

## Introduction

Sjögren syndrome (SS) is a chronic inflammatory disease that affects the exocrine glands, particularly the salivary and lacrimal glands. Gland histology is typically characterized by focal mononuclear cell infiltrates surrounding the glandular ducts, which, in some cases, replace the secretory units [1], [2]. The prevalence of SS in the general population is approximately 0.1–0.4%, which is comparable to that of rheumatoid arthritis, although SS has a high female to male ratio of 9:1 [3], [4], [5]. The disease is also characterized by the presence of epithelial cell apoptosis, pathogenic autoantibodies, and overexpression of type I interferon (IFN) [6], [7], [8], which can lead to exocrinopathy and extraglandular organ damage. Although the exact pathogenetic mechanisms are not yet fully elucidated, one of the possible mechanisms is increased ductal epithelial cell apoptosis, which can trigger a complex inflammatory cascade [6], [9].

Growth arrest-specific protein 6 (Gas6) is the most recently identified member of the family of plasma vitamin K-dependent proteins. It was cloned and characterized in 1993 and found to be similar to plasma anticoagulant protein S [10]. Gas6 also resembles a growth factor-like molecule, as it interacts with receptor tyrosine kinases of the TAM (Tyro–3, Axl, and Mer) family [11]. The Gas6/TAM system has been found to be important in inflammation, hemostasis, cancer, and autoimmune diseases [12], [13]. It has also previously been shown that preincubation with Gas6 can decrease the secretion of proinflammatory cytokines from dendritic cells, and that Gas6 plays an important role in the clearance of apoptotic cells by macrophages and dendritic cells [14], [15]. TAM triple knockout mice display profound lymphoproliferation and features of systemic autoimmunity [16]. Studies of minor salivary gland (MSG) tissues in SS reveal that salivary gland epithelial cells (SGEC) express a plethora of immune-competent molecules implicated in innate and acquired immune responses [17], [18]. Among the pathways implicated in SS pathogenesis, elevated in-situ apoptosis of SGEC and the release of autoantigen-containing apoptotic blebs, as well as the secretion of exosomes that are involved in the transfer of antigens to antigen-presenting cells, are hypothesized as the initial insult of SS [19], [20], [21], [22], [23]. Indeed, SGEC have been shown to constitutively release exosomes, which contain the autoantigenic proteins Ro/SS-A and La/SS-B that are major targets of SS [24]. The central role of the epithelium in SS pathogenesis justifies the renaming of the disease to “autoimmune epithelitis,” which emphasizes an immunoregulatory role in the development and perpetuation of disease [17], [25], [26]. Recent reports indicate that the Gas6/TAM system is also involved in the pathogenesis of systemic lupus erythematosus (SLE), and that Gas6 may correlate in several ways with disease activity in SLE [27], [28], [29]. As part of the category of autoimmune diseases like SLE, SS reveals an over-expression of type I IFN-inducible genes in the peripheral blood and salivary glands. We therefore hypothesized that the Gas6/TAM system might share a similarly important role in the pathogenesis of SS. In order to address this issue, we assessed plasma Gas6 levels and protein expression in labial salivary gland (LSG) tissues from SS patients.

## Materials and Methods

### Ethics statement

This study was approved by the institutional review board (IRB) of the Tri-Service General Hospital (TSGHIRB No.:2-101-05-107). Informed consent was obtained from each subject in person prior to participation in the study. All the study participants provided signed and informed consent, and this process was documented in an IRB-approved consent form. The consent procedure was approved by the Ethics Committee of TSGH. This study was conducted in accordance with the principles expressed in the Declaration of Helsinki.

### Individuals enrolled in the study

A total of 254 adults were recruited from the outpatient clinics of the Tri-Service General Hospital, Taipei, Taiwan. Plasma samples from a group of patients with SS (n = 193) were obtained from the Division of Rheumatology, Allergy, and Immunology. Samples from a group of healthy individuals (n = 61) without autoimmune disease, coagulopathy, malignant tumor history, or infection within recent weeks, and not taking any anticoagulant or antidiabetic medication, were also obtained. The two groups were matched for ethnicity (Han Chinese), age, body weight, and gender. Patients with SS fulfilled the revised criteria of the American-European Consensus Group for the classification of SS [30]. According to the revised rules for classification, primary SS (pSS) was defined in 159 patients without any potentially associated disease. Patients with a potentially associated disease, such as another well-defined connective tissue disease, were considered to have secondary SS (sSS). Thirty-four subjects with sSS, associated with systemic lupus erythematosus (SLE) (n = 20), rheumatoid arthritis (RA) (n = 9), primary biliary cirrhosis (n = 4), and systemic sclerosis (n = 1) were recruited sequentially. All blood samples were stored at −80°C immediately after collection. Information on patient medical history, physical examination, and clinical symptoms were registered by a rheumatologist prior to plasma sampling. For each patient, the blood cell count, routine chemistry, urinalysis, anti-SSA (Ro) antibody, anti-SSB (La) antibody, IgG, IgA, IgM, C3, and C4 were assessed. Anti-SSA and anti-SSB antibodies were measured by radioimmunoassay using a commercially available kit (Thermo Fisher Scientific- Phadia GmbH, MA, USA). Body mass index (BMI) was calculated as weight in kilograms divided by the square of height in meters and was expressed using established guidelines [31].

### Measurement of Gas6

Plasma Gas6 protein was measured using the DuoSet^®^ enzyme-linked immunosorbent assay (ELISA) Development kit (R&D Systems, Minneapolis, MN, USA), which contains the basic components required for the development of a sandwich ELISA to measure natural and recombinant human Gas6. For each plasma sample, 100 μl was directly transferred to the microtest strip wells of the ELISA plate coated with capture antibody of mouse anti-human Gas6 and incubated for 2 h at room temperature. After three subsequent washing steps, the detection antibody was added, and the reaction mixture was incubated for 2 h at room temperature. Antibody binding was detected with streptavidin-conjugated horseradish peroxidase and developed with a substrate solution. The reaction was stopped with a stop solution, and optical density was determined using a microplate reader set at 450 nm. The Gas6 concentration was quantitated by a calibration curve using a human Gas6 standard. Each plasma sample was assayed in duplicate according to the instructions of the manufacturers, and the values were within the linear portion of the standard curve. This technique has been validated previously with intra-assay and inter-assay coefficients of variation (CVs) for Gas6 reported as 6.5 and 8.5%, respectively, with a mean recovery in 10 patients of 97%, and a lower limit of quantification of 0.26 ng/ml [32].

### Gas6 expression in LSG

Human LSG tissues were excised by the oral surgeon and frozen immediately at -80°C. The snap-frozen tissues were embedded with Optimal Cutting Temperature (OCT) medium (Surgipath, #01480; Leica Biosystems Nussloch GmbH, Nussloch, Germany) and equilibrated at -20°C for sectioning at 5 mm. Sections were air-dried for 5 min and washed to remove OCT. For immunohistochemical staining, each section was blocked with blocking solution for 1 h and incubated for 15 min in 3% H_2_O_2_ that was diluted in methanol, with complete washing of the sections between steps. Specimens of the LSG were stained with goat antibody polyclonal to Gas6 (R&D Systems, #AF885) that was diluted in Dako diluent (Dako Denmark A/S, Glostrup, Denmark, #s3022) for 1 h at room temperature, followed by detection with the Dako REAL EnVision system (Dako Denmark A/S, #K5007), and mounting under cover slips. Immunostaining validation is confirmed by western blot and staining analysis in epithelial cell lines with/without GAS6 plasmid transfection (S1 Fig). To assess immune-reactivity in histologic sections, all tissue experiments were repeated twice and the histologic slides were checked & scored by two investigators (LCH and PYJ) concurrently who did not have prior knowledge of patients’ clinical status. To evaluate Gas6 immunostaining scores, the intensity of cytoplasmic and membranous staining was scored as 0 (absence of staining), 1 (weak staining), 2 (moderate staining), or 3 (strong staining) (S2 Fig). Weak, moderate, and strong cytoplasmic stainings were identified by microscopy with magnification of 40×, 20×, and 10×, respectively. The cut-off value of Gas6 intensity scores was set by the magnification of microscope objective lens.

### Statistical methods

Statistical analysis was performed using SPSS software, version 13.0 (SPSS, Chicago, IL, USA). Normal distributions and homogeneity of variances were evaluated using Levene’s test, and non-normally distributed variables were log-transformed if necessary. A *p*-value < 0.05 was regarded as statistically significant. Data were expressed as the mean ± standard deviation (SD). The difference in Gas6 levels between SS patients and normal controls was determined using an independent t-test. The white blood cell (WBC) count, hemoglobin (Hb) level, platelet count, anti-SSA level, anti-SSB level, and Gas6 level were analyzed and tested for significance on a log scale. Multivariate linear regression analysis was used to evaluate the independent determinants of plasma Gas6 and other covariates. Data were expressed as odds ratio (95% CI). The Gas6 immunostain scores were calculated by unpaired t test.

## Results

### Characteristics of the study population

Tables 1 and 2 summarize the main demographic data of the study population. SS patients and controls had comparable ages (53.4 ± 10.2 *vs*. 52.0 ± 9.0 years), frequencies of male gender (7.8 *vs*. 13.1%), body weight (56.5 ± 9.3 *vs*. 57.6 ± 10.8 kg), and BMI (22.6 ± 3.4 *vs*. 22.8 ± 3.7 kg/m^2^). The SS patients had a mean ± SD disease duration of 5.2 ± 2.3 years. Plasma Gas6 levels, WBC counts, and lymphocyte counts of SS patients were lower than those in normal controls (13.6 ± 9.1 *vs*. 19.9 ± 13.4 ng/mL, *p* < 0.001; 5.6 ± 1.6 *v*s. 6.4±1.7 x 10^3^/μL, *p* < 0.005; and 1.37 ± 0.70 *vs*. 1.97 ± 0.73 x 10^3^/μL, *p* < 0.001, respectively). When SS patients were divided into the pSS and sSS groups, the Gas6 levels of pSS patients were lower than those of normal controls (13.5 ± 8.6 *vs*. 19.9 ± 13.4 ng/mL, *p* < 0.001). There were, however, no significant differences in Gas6 levels between sSS patients and normal controls (16.9 ± 11.2 *vs*. 19.9 ± 13.4 ng/mL, *p* = 0.068). Additionally, an association analysis was performed to evaluate whether seropositivity (anti-SSA and anti-SSB) correlated with Gas6 in SS patients. The Gas6 levels in the seropositive group (n = 155) and seronegative group (n = 38) were not statistically different (14.1 ± 9.4 *vs*. 11.6 ± 7.6 ng/mL, *p* > 0.05). The mean age of the seronegative group was, however, higher than that of the seropositive group (56.9 ± 7.8 *vs*. 51.4 ± 10.4 years, *p* < 0.005), while in the seropositive group, the WBC and lymphocyte counts were lower than those in the seronegative group (5.5 ± 1.6 *vs*. 6.1 ± 1.7 x 10^3^/μL, *p* < 0.05, and 1.29 ± 0.68 *vs*. 1.97 ± 0.58 x 10^3^/μL, *p* < 0.01, respectively, Table 3).

**Table 1:** Anthropometric and biochemical variables among Sjögren syndrome.

	Normal (n = 61)	Sjögren syndrome (n = 193)	*P*
Age (yrs)	52.0±9.0	53.4±10.2	ns
Sex (male, %)	13.1%	7.8%	ns
Disease duration (yrs)	NA	5.2±2.3	
Body weight (kg)	57.6±10.8	56.5±9.3	ns
BMI (kg/m^2^)	22.8±3.7	22.6±3.4	ns
WBC (x 10^3^/μL)	6.4±1.7	5.6±1.6	<0.005
Hb (g/dL)	13.2±1.5	12.9±1.2	ns
Platelet (x 10^3^/μL)	225.3±52.6	210.2±52.3	ns
Neutrophil (x 10^3^/μL)	3.85±1.50	3.55±1.76	ns
Lymphocyte (x 10^3^/μL)	1.97±0.73	1.37±0.70	<0.001
Ro/La(+)	ND	80.3%	
IgG (mg/dL)	1318.1±235.8	1259.3±321.3	ns
IgA (mg/dL)	274.3±80.6	266.6±90.9	ns
IgM (mg/dL)	113.7±48.3	112.9±71.8	ns
C3 (mg/dL)	98.5±16.9	90.4±31.9	ns
C4 (mg/dL)	20.9±6.0	18.6±11.1	ns
Gas6 (ng/ml)	19.9±13.4	13.6±9.1	<0.001

Data were shown as mean±SD

Abbreviation: BMI: body mass index; WBC: white cell count; Hb: hemogloblin; Gas6: growth-arrest specific protein 6; ND: no done; NA: no applicable; ns: not significant

**Table 2:** Anthropometric and biochemical variables between primary and secondary Sjögren syndrome.

	Normal (n = 61)	Primary SS (n = 159)	Secondary SS (n = 34)	*P* ^*1*^	*P* ^*2*^	*P* ^*3*^
Age (yrs)	52.0±9.0	53.5±9.7	47.7±11.1	ns	ns	<0.005
Sex (male, %)	13.1%	6.9%	11.8%	ns	ns	ns
Disease duration (yrs)	NA	4.7±2.4	5.7±2.2	NA	NA	ns
Body weight (kg)	57.6±10.8	56.5±8.4	56.4±12.9	ns	ns	ns
BMI (kg/m^2^)	22.8±3.7	22.7±3.1	22.1±4.5	ns	ns	ns
WBC (x 10^3^/μL)	6.4±1.7	5.6±1.6	5.5±1.7	<0.005	<0.05	ns
Hb (g/dL)	13.2±1.5	13.0±1.2	12.7±1.4	ns	ns	ns
Platelet (x 10^3^/μL)	225.3±52.6	211.2±51.7	205.0±55.8	ns	ns	ns
Neutrophil (x 10^3^/μL)	3.85±1.50	3.94±1.64	3.72±2.15	ns	ns	ns
Lymphocyte (x 10^3^/μL)	1.97±0.73	1.67±0.75	1.10±0.54	ns	<0.001	<0.005
Ro/La(+)	ND	78.6%	90.9%			ns
IgG (mg/dL)	1318.1±235.8	1369.5±306.7	1173.2±310.2	ns	ns	<0.05
IgA (mg/dL)	274.3±80.6	279.5±92.2	255.7±89.4	ns	ns	ns
IgM (mg/dL)	113.7±48.3	108.2±66.1	117.2±77.5	ns	ns	ns
C3 (mg/dL)	98.5±16.9	103.9±29.4	78.9±29.7	ns	<0.005	<0.005
C4 (mg/dL)	20.9±6.0	20.9±9.4	16.6±12.2	ns	ns	ns
Gas6 (ng/ml)	19.9±13.4	13.5±8.6	16.9±11.2	<0.001	ns	0.068

*P* ^*1*^: normal vs. pSS; *P* ^*2*^: normal vs. sSS; *P* ^*3*^: pSS vs. sSS

Data were shown as mean±SD

Abbreviation: BMI: body mass index; WBC: white cell count; Hb: hemogloblin; Gas6: growth-arrest specific protein 6; pSS: primary Sj**ö**gren syndrome; sSS: secondary Sj**ö**gren syndrome; ND: no done; NA: no applicable; ns: not significant

**Table 3:** The difference between Sjögren syndrome with Ro/La (+) and Ro/La (-).

	Ro/La (+) (n = 155)	Ro/La (-) (n = 38)	*P*
Age (yrs)	51.4±10.4	56.9±7.8	<0.005
Body weight (kg)	56.4±9.8	56.6±6.8	ns
BMI (kg/m^2^)	22.6±3.5	22.8±2.9	ns
WBC (x 10^3^/μL)	5.5±1.6	6.1±1.7	<0.05
Hb (g/dL)	13.0±1.2	13.2±1.2	ns
Platelet (x 10^3^/μL)	207.0±46.3	227.7±72.5	ns
Neutrophil (x 10^3^/μL)	3.45±1.80	3.91±1.62	ns
Lymphocyte (x 10^3^/μL)	1.29±0.68	1.97±0.58	<0.01
Gas6 (ng/ml)	14.1±9.4	11.6±7.6	ns

Data were shown as mean±SD

Abbreviation: BMI: body mass index; WBC: white cell count; Hb: hemogloblin; Gas6: growth-arrest specific protein 6; ns: not significant

The results of a multivariate logistic regression analysis to investigate whether plasma Gas6 values were related to SS, independently of other confounding factors, are shown in Table 4. After adjustment for WBC count, Hb level, and platelet and lymphocyte counts, every decrease of 1 ng/mL in plasma Gas6 concentration was related to a 5% and 8% increase in the risk of development of SS (SS *vs*. normal controls, 0.95 [0.90–0.99]) and pSS (pSS *vs*. normal controls, 0.92 [0.86–0.99]), respectively. This association remained significant after further adjustment for other covariates, including C3 and C4 levels (SS *vs*. normal controls, 0.94 [0.90–0.99]; pSS *vs*. normal controls, 0.92 [0.86–0.99]) (all *p* < 0.05).

**Table 4:** Multivariate linear regression analyses of plasma Gas6 concentration among Sjögren syndrome.

	SS vs normal		pSS vs normal		sSS vs normal	
	OR	95%CI	OR	95%CI	OR	95%CI
model I	0.95	(0.93~0.98)*	0.94	(0.91~0.97)*	0.98	(0.94~1.01)
model II	0.95	(0.90~0.99)**	0.92	(0.86~0.99)**	0.98	(0.92~1.01)
model III	0.94	(0.90~0.99)***	0.92	(0.86~0.99)***	0.98	(0.91~1.02)

model I: crude

model II: adjusted for WBC, Hb, platelet and lymphocyte counts

model III: further adjusted for C3 and C4

*p<0.001;

**p<0.01;

***p<0.05

### Immunohistochemical staining of Gas6 of LSG

**Fig 1:**
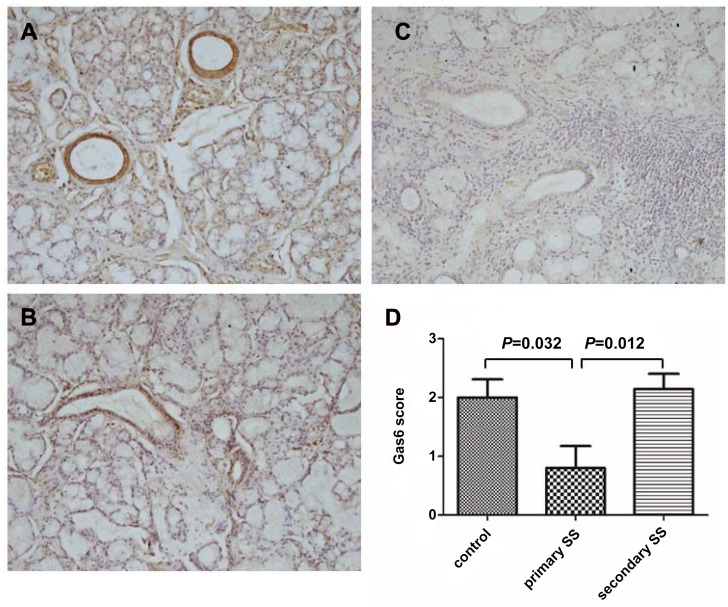
Immunohistochemical staining of Gas6 in salivary gland tissues from normal controls, primary and secondary Sjögren syndrome patients. Immunohistochemical staining of Gas6 in normal control tissue (A); secondary Sjögren syndrome (B); and primary Sjögren syndrome (C). Original magnification ×400; and (D) The ductal immunostain scores in control, secondary Sjögren syndrome and primary Sjögren syndrome were statistically analyzed. The *p* value was calibrated between each group by unpaired t test.

In this study, we examined the distribution and expression of Gas6 in the LSG by immunohistochemical staining. The results revealed most positive staining for Gas6 in the intercalated, striated, and interlobular ducts of LSG, but variably in the acinar cells and immune infiltrates (Fig 1A–1C). The ductal immunostain scores were statistically lower in pSS patients compared with normal controls (*p* = 0.032) and sSS patients (*p* = 0.012) (Fig 1D).

## Discussion

Numerous studies have shown that the Gas6/TAM system regulates cell proliferation, migration, adhesion, phagocytosis, and survival. Consequently, altered activity/expression of Gas6/TAM components have been discovered in a variety of pathologies, such as inflammation, coagulopathy, cancer, diabetic vascular and renal diseases, and autoimmune disease [12], [13]. However, direct clinical evidence as to whether the Gas6/TAM system is involved in SS is still lacking. Our results, described for the first time herein, revealed that plasma Gas6 concentrations and Gas6 expressions in LSG were decreased in patients with SS, particularly in patients with pSS. A growing body of evidence indicates that glandular and acinar epithelial inflammation and activation of the innate immune system at disease sites are closely involved in the pathogenesis of SS [33], [34]. Hence, based on the present findings, we speculate that Gas6/TAM signaling may play a potential role in the pathogenesis of SS. Previous reports have also shown Gas6/TAM signaling and resultant intrinsic inhibition of inflammatory responses in dendritic cells and macrophages, indicating a potential role for the Gas6 protein in controlling innate immunity and inflammation processes [16]. Otherwise, TAM triple knockout mice with low Gas6 levels show hyperactivation of monocytes/macrophages, and their monocytes react with an excessive secretion of tumor necrosis factor-α and interleukin–6 after lipopolysaccharide challenge [35]. In patients with pSS, infiltration of the MSG by macrophages and dendritic cells appears to play an active role in the expansion and organization of gland infiltrative injuries [36]. We speculated that the inflammatory cascade of SS may be, at least in part, mediated through low plasma Gas6 levels, as well as glandular Gas6 expression and, consequently, activated innate immunity.

SS can occur alone, which is defined as pSS, or in combination with almost any other autoimmune disease, most frequently SLE and RA, which is then defined as sSS. Recent studies reported the Gas6/TAM system was involved in the pathogenesis of SLE, and was also associated with disease activity in SLE. As elevated plasma concentrations of Gas6 vary with disease activity in SLE and may have a role in lupus pathogenesis, Gas6 may be useful as a biomarker of SLE disease activity [27], [28], [29]. However, other studies also point to a clear association between reduced Gas6 levels and SLE [37], [38]. The conflicting nature of these reports may, however, be explained by the variation in disease status of study-enrolled patients. More recently, a decreased Gas6 level in RA was reported by Iman et al. [39]. Insufficient apoptosis of synovial macrophages, fibroblasts, and lymphocytes is proposed as one mechanism that might contribute to the persistence of RA. Gas6 has been shown to be required for the efficient uptake of apoptotic cells by macrophages, and decreased Gas6 levels may lead to an inefficient clearance of apoptotic cells, resulting in the exposure of cellular contents to immune cells and the propagation of an inflammatory response. Overall, unlike in pSS patients, the Gas6 level in sSS patients was not significantly different from that in normal controls. Our explanation is that SLE was the major associated disease in sSS in the present study, and higher Gas6 levels in these categories of patients could have contributed to the lack of statistical difference observed between sSS and normal subjects. Alternatively, several confounding factors could have influenced the level of Gas6, such as age, gender, glucose status, and body weight, which may have affected the statistical significance of the difference between levels in sSS patients and normal controls [40].

Leukopenia is the second most reported hematological abnormality in pSS. A previous study reported a close association between leukopenia and positive autoantibodies (mainly anti-SSA and anti-SSB antibodies) [41]. The differential leukocyte count has been studied in large series of pSS patients. The most frequent abnormality was lymphopenia, followed by neutropenia. In pSS, lymphopenia has been associated with a higher prevalence of autoantibodies [42]. This association was confirmed in our study (Table 3). The causes of leukopenia in SS likely include an antibody-mediated accelerated turnover of developing leukocytes, splenic trapping, suppression of early cellular proliferation, and possible accelerated apoptosis. However, our results did not show any differences in Gas6 levels between the seronegative and seropositive groups, indicating that the antibody-mediated mechanism may play a more etiopathogenic role in SS patients with leukopenia.

To the best of our knowledge, no *in vitro* or *in vivo* studies to date have explored the role of the Gas6/TAM system in SS. The strength of our clinical study revealed a novel, strong association between Gas6 levels in blood and salivary tissue and SS, particularly pSS. Nevertheless, some limitations of the present study remain to be addressed: first, this study was not aimed at elucidating pathophysiological pathways of Gas6 in SS, but was rather designed to evaluate its possible clinical significance. Second, our study was purely observational and the possible physiological effects of plasma Gas6 were beyond the scope of our study. Third, the TAM receptors (Tyro–3, Axl, and Mer) in SS patients were not assayed, precluding the adequate interpretation of Gas6/TAM interactions in SS. Our preliminary report indicated no strong relationship between pSS disease activity and plasma Gas6 concentrations. Future studies should consider correlations of the European League Against Rheumatism (EULAR)-SS disease activity index (ESSDAI) with the entire Gas6/TAM system. To further clarify the significance and mechanisms underlying the observed differences in SS patients, as well as the potential clinical consequences, more research to explore the roles of the Gas6/TAM system in SS patients is needed.

We conclude that decreased plasma Gas6 levels and its expression in LSG are associated with pSS. Additionally, these findings support the hypothesis that modulation of Gas6 activity may provide an important target for interventions in pSS. Gas6/TAM signaling may represent a new class of therapeutic targets in autoimmune disease like SS. Understanding the nature of the Gas6/TAM interaction would ultimately aid in the development of novel drugs for therapeutic applications in SS, where interactions between Gas6 and TAM receptors contribute to disease progression or pathology.

## Supporting Information

S1 Fig Validation of immunohistochemical staining of Gas6. Validation of immunohistochemical staining of Gas6. (A) Oral squamous cell carcinoma cells transfected with Gas6 plasmid overexpress Gas6, confirmed by western blot. (B) Cells were fixed in 10% paraformaldehyde for 10 min, washed in PBS and then incubated in PBS containing 2% bovine serum albumin (BSA) for another 10 min. Next, cells were blocked with blocking solution for 1 h and cells were stained with goat polyclonal to Gas6 (R&D System, Inc., #AF885) that was diluted in Dako diluent (Dako Denmark A/S, Glostrup, Denmark, #s3022) for 1 h at room temperature, and this was followed by detection with the Dako REAL EnVision system (Dako Denmark A/S, #K5007) and mounting under cover slips. Some of transfected cells overexpress cytoplasmic Gas6 protein immunocytochemically. The methodology is the same as immunohistochemistry staining of Gas6 expression in human salivary gland tissues.
(TIF)

S2 Fig Scores of immunohistochemical staining of Gas6. The intensity of cytoplasmic and membranous staining was scored as 0 (absence of staining), 1 (weak staining), 2 (moderate staining), or 3 (strong staining).
(TIF)
