# Supplementary figures and images for: Growth Arrest-Specific 6 Protein in Patients with Sjögren Syndrome: Determination of the Plasma Level and Expression in the Labial Salivary Gland
Source: PLoS One. 2015 Oct 7;10(10):e0139955. doi: 10.1371/journal.pone.0139955 (PMC4596882; doi:10.1371/journal.pone.0139955)

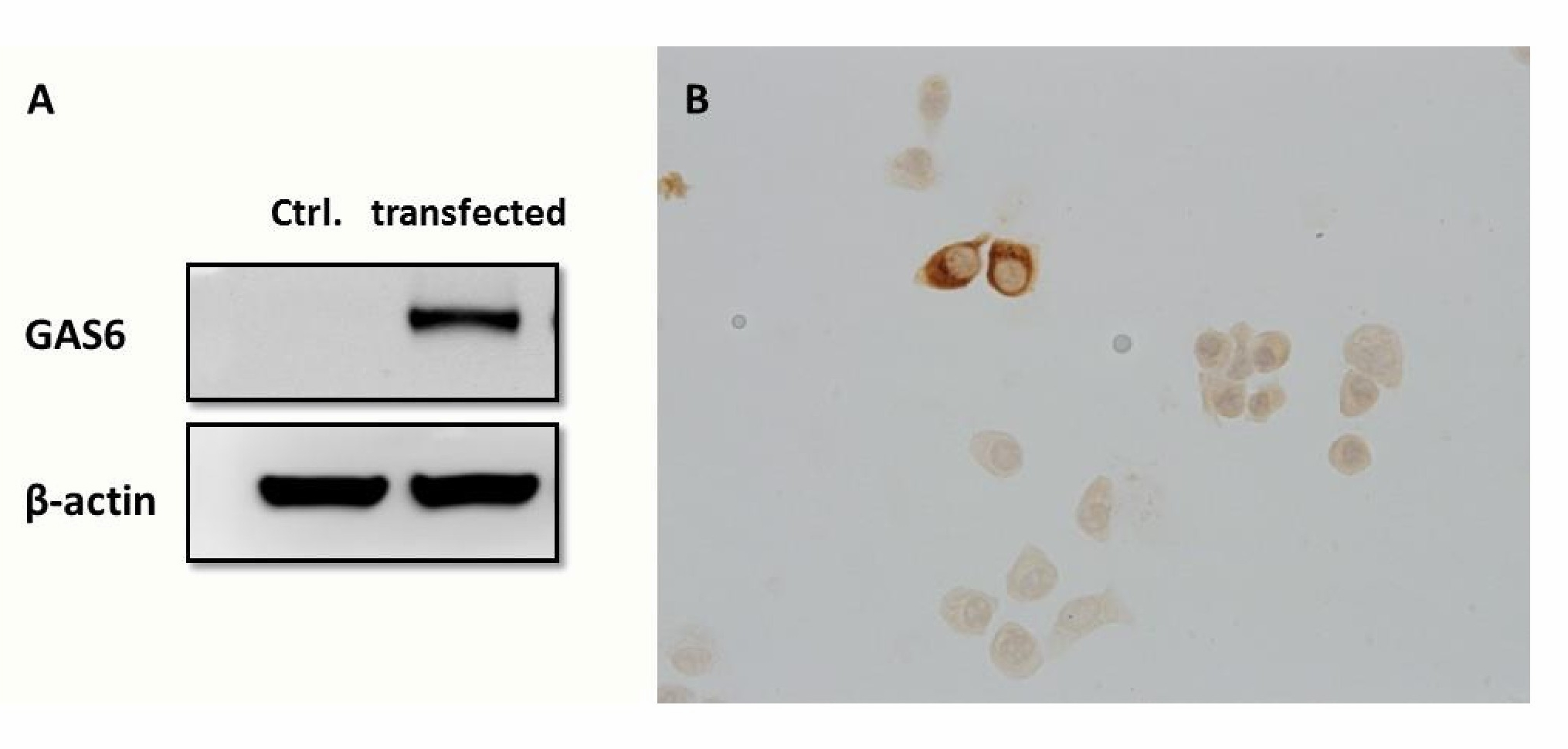

Supplement: S1 Fig — Validation of immunohistochemical staining of Gas6. (A) Oral squamous cell carcinoma cells transfected with Gas6 plasmid overexpress Gas6, confirmed by western blot. (B) Cells were fixed in 10% paraformaldehyde for 10 min, washed in PBS and then incubated in PBS containing 2% bovine serum albumin (BSA) for another 10 min. Next, cells were blocked with blocking solution for 1 h and cells were stained with goat polyclonal to Gas6 (R&D System, Inc., #AF885) that was diluted in Dako diluent (Dako Denmark A/S, Glostrup, Denmark, #s3022) for 1 h at room temperature, and this was followed by detection with the Dako REAL EnVision system (Dako Denmark A/S, #K5007) and mounting under cover slips. Some of transfected cells overexpress cytoplasmic Gas6 protein immunocytochemically. The methodology is the same as immunohistochemistry staining of Gas6 expression in human salivary gland tissues. (TIF) [file pone.0139955.s001.tif]

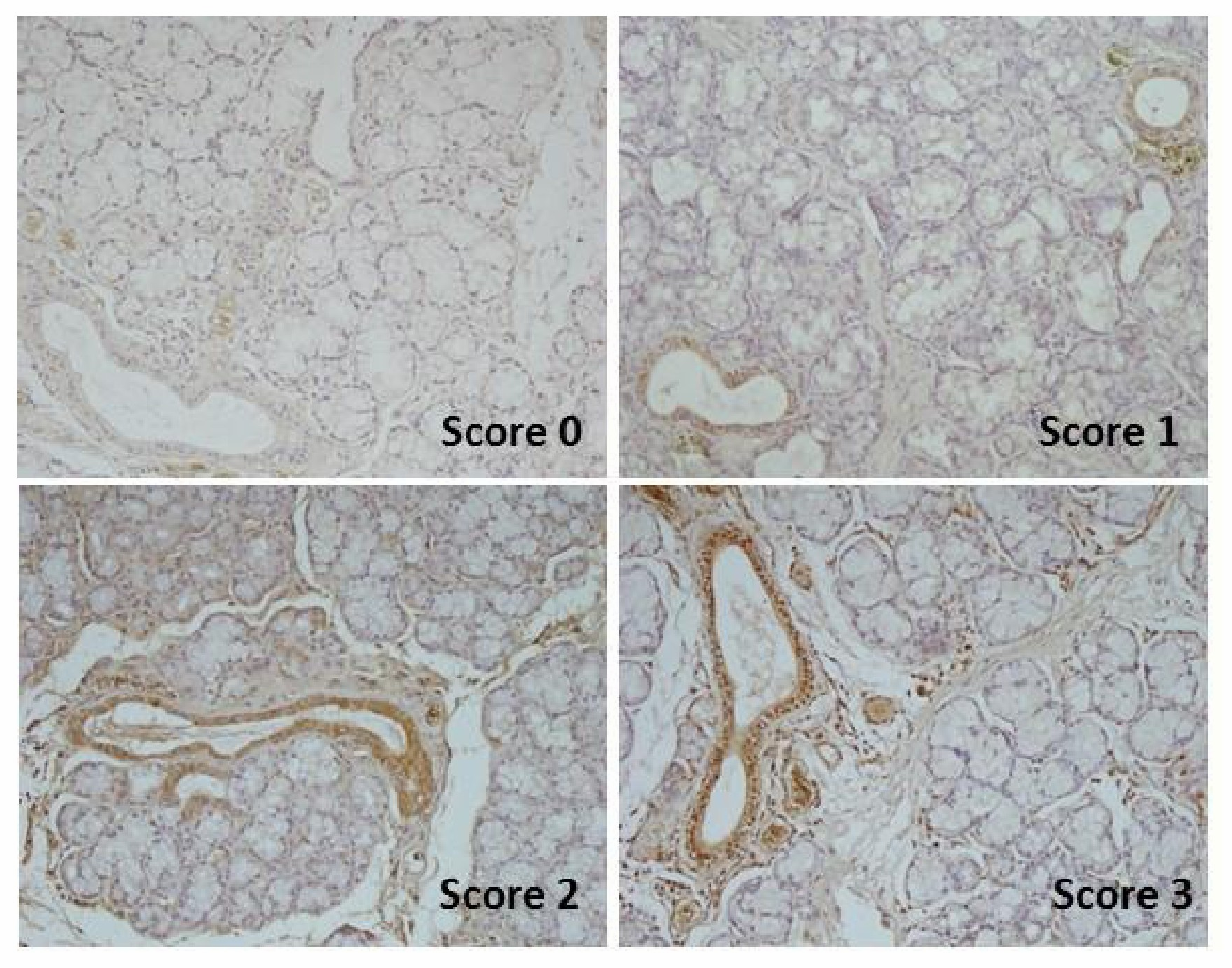

Supplement: S2 Fig — The intensity of cytoplasmic and membranous staining was scored as 0 (absence of staining), 1 (weak staining), 2 (moderate staining), or 3 (strong staining). (TIF) [file pone.0139955.s002.tif]
